# Relationship between front-of-pack labeling and nutritional characteristics of food products: An attempt of an analytical approach

**DOI:** 10.3389/fnut.2022.963592

**Published:** 2022-08-19

**Authors:** Daniela Martini, Franca Marangoni, Alessandro Banterle, Lorenzo Maria Donini, Gabriele Riccardi, Andrea Poli, Nicoletta Pellegrini

**Affiliations:** ^1^Department of Food, Environmental and Nutritional Sciences (DeFENS), Università degli Studi di Milano, Milan, Italy; ^2^Nutrition Foundation of Italy, Milan, Italy; ^3^Department of Environmental Science and Policy (ESP), Università degli Studi di Milano, Milan, Italy; ^4^Department of Experimental Medicine, Sapienza University, Rome, Italy; ^5^Department of Clinical Medicine and Surgery, Federico II University, Naples, Italy; ^6^Department of Agricultural, Food, Environmental and Animal Sciences, University of Udine, Udine, Italy

**Keywords:** front-of-pack labeling (FOPL), NutrInform Battery, Nutri-Score, Keyhole, nutrient composition, nutrition information, overall balanced diet

## Abstract

The adoption of supplementary nutrition information, i.e., front-of-pack labeling (FOPL), on pre-packed food products is advocated as a tool to improve the consumers' knowledge of the nutrient content or the nutritional quality of foods, but also to drive products reformulation by the food industry. Ultimately, FOPL should help people to select foods in order to compose an overall balanced diet, which is essential for health. However, the extent to which the different FOPL systems proposed in the European Union (EU) (interpretative or informative) are effectively able to convey the information useful to improve both food choices and dietary habits of the consumers is still under debate and needs to be analyzed in detail. The use of 3 FOPL schemes proposed within the EU (Nutri-Score, Keyhole and NutrInform Battery) to compare products available on the Italian market within different food categories, highlights some critical issues: (1) different FOPL provide to consumers different kinds of information; (2) systems based on similar theoretical approaches can provide conflicting information; (3) the algorithms on which interpretative FOPL are based can give the same summary information for products differing in nutrient composition, impact on the overall dietary balance and therefore on the health of people with different characteristics, physiological/pathological conditions, and nutritional requirements; (4) on the other hand, products with similar nutrient composition can obtain different interpretative FOPL; (5) informative systems are generally more complex and require greater both attention and knowledge from the consumer; (6) FOPL based on 100 g of product overlook the role of portion (and frequency of consumption) in determining the nutrient intake without informing on the contribution of a single food to the overall diet; (7) FOPL based on scoring systems could promote the reformulation of selected products, especially with a composition very close to the threshold limits; (8) for the portion-based informative FOPL systems, the incentive for reformulation could essentially involve the reduction of portion size. Finally, the importance of nutritional education interventions, which are required to encourage the use by consumers of informative FOPL systems, cannot be neglected to improve the quality of diets regardless of the FOPL used.

## Introduction

The interest in nutrition labeling as a policy tool potentially useful to promote healthy diets has increased in last years, mainly due to the large diffusion among the population of noncommunicable diseases, which are in part related to diet (1).

The relationship between diet and health has in fact been confirmed by the most recent scientific research: according to the Global Burden of Disease study group, which analyzed 286 causes of death and 87 risk factors in about 200 countries and territories of the world, diet-related risk factors were altogether responsible, in 2019, for 13.5 and 14.6% of all female and male deaths, respectively (about 46% of cardiovascular disease deaths and 8% of cancer deaths in Countries with high socio-demographic index) (2, 3). The demographic changes that are taking place in the European population, and mainly the progressive increase in the average age, as well as in life expectancy at birth, together with the increasing prevalence of age-related risk factors, make the diet-health association even more important, and complex to interpret (4). In this context, all the tools which may prove useful to improve the nutritional information of the general population, in order to enable consumers to make healthier food choices, are gaining importance.

In particular, front-of-pack labeling (FOPL), which is a form of supplementary nutrition information, is increasingly considered not only a tool to drive reformulation by the food industry, but also an effective strategy to improve the consumers' knowledge and awareness of the nutrient content or nutritional quality of the food. Such result may be achieved by helping consumers to better understand the nutrition declaration, which is included in the list of mandatory information (together with the list of ingredients, net quantity of the food, date of minimum durability, any special storage conditions and/or conditions of use, name and address of the food business operator, country of origin or place of provenance), the actual comprehension of which may be limited (5).

According to the Regulation (EU) n. 1169/2011 (art. 35), energy value and nutrient content, which are already reported in the back of pack labeling, may be also presented by other forms of expression and/or using graphical forms or symbols in addition to words or numbers, with the aim “to facilitate consumer understanding of the contribution or importance of the food to the energy and nutrient content of a diet”. This goal, together with the proposal to harmonize mandatory front-of-pack nutrition labeling in the EU, is picked up by the Farm to Fork strategy “to empower consumers to make informed, healthy and sustainable food choices” (6).

The aim of this paper is to propose a critical assessment of the role of FOPL schemes in providing nutrition information useful to improve both consumer choices and dietary habits, which are essential to favorably affect public health.

## Characteristics of FOPL

Various schemes of FOPL have been developed in the last 40 years, of which the most prevalent in the European Union are those based on the guideline daily amount (GDA) concept, on a traffic light scheme or on qualifying (or disqualifying) threshold criteria (7): the main characteristics of Nutri-Score and Keyhole based on the latter approach and of NutrInform Battery, which is based on the first one, are summarized in Table 1.

**Table 1:** Main characteristics of three FOPL schemes.

	**NutrInform Battery**	**Nutri-Score**	**Keyhole**
Logo	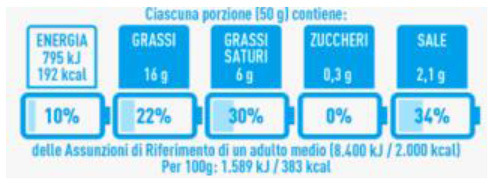	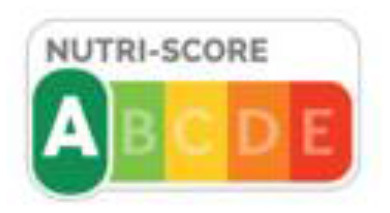	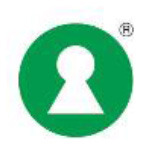
Type of scheme	Informative	Interpretative	Interpretative (supportive?)
Based on	Reference Intakes	Algorithm (nutrient thresholds)	Nutrient thresholds
Information included (positive or negative)	Neutral: energy, total fat, saturated fat, sugar, salt	Negative: energy, saturated fat, sugar, salt. Positive: fiber, protein, vegetables, fruit, legumes, olive/nut/canola oils	Less salt, less sugar, more fiber, more whole grains and healthier fat
Main declared purpose	To make the mandatory nutritional information pursuant to Reg. (EU) no. 1169/2011 more easily comprehensible for the consumer	To inform about the nutritional quality of a product	To help consumers identify the healthier options when buying food
Amount of food	Portion	100 g	100 g
Contribution to the whole diet	Yes	No	No
Food categories	Across-the-board	Across-the-board (exceptions)	Category specific

The Reference Intakes label provides numerical information on the amount of energy and of some nutrients present in a portion of food and the percentage contribution to the daily reference intake for calories and nutrients set out in Annex XIII of the Regulation (EU) n. 1169/2011; the NutrInform Battery, proposed by the Italian government and notified to the European Commission in January 2020, which is based on this principle, is an example of informative FOPL scheme (8).

The interpretative approach has been the basis for schemes aimed at classifying foods according to the content of a limited number of single nutrients, based on a traffic light scheme, or to the whole nutrient profile assessed by algorithms, again based on the content of a limited number of single nutrients or ingredients. Nutri-Score, the FOPL used in France since 2017, is an example of the interpretative approach (9): it is a summary indicator of the nutritional quality of a product along a graded scale presented in ordered colors (from dark green to dark orange) and letters (A–E). The same approach is at least in part shared by the Multiple Traffic Light (MTL) system, which is recommended by UK Health Ministers since 2013 on a voluntary basis, to show consumers if the prepackaged food has low, medium or high amounts of fat, saturated fat, sugars and salt per 100 g; however, in this kind of FOP the same information is reported even per portion as both absolute amounts and percentage of adult's reference intakes (10).

Furthermore, threshold criteria may be applied to define endorsement and summary logos or warning label on foods: in the first case, as for the Nordic Keyhole, which was first introduced in Sweden in 1989, the logo certifies that a product meets certain requirements for nutrient content in a category-based nutrient profile model (it is set for 32 food groups and registered as a trademark by the Swedish Food Agency) (11); warning (or negative) FOPL, as in the Chile experience and used in Finland for salt, the food package must bear a warning symbol if the set thresholds are exceeded (12).

From a practical point of view, these are two substantially different approaches: one (the informative one) more complex is aimed at providing more information to the consumer (i.e., schemes based on reference intakes), while the other (the interpretative) is more concise and aimed at simplifying the consumers' choices, synthesizing a number of information into a single one (13).

It is important to underline that while for informative systems, which simply intend to provide information on some selected food composition aspects, efficacy validation is required only with regard to the correct understanding of the conveyed information by the consumer, for the interpretative systems, which are based on specific algorithms, built to integrate the information related to the content of different nutrients and ingredients in a single parameter, adequate experimental support is required that confirms their validity in actually improving the nutritional quality of the diet of consumers exposed to this FOPL. The criteria on which these algorithms are based are in fact characterized by a wide discretion regarding the weight attributed to the individual nutrient considered, the thresholds adopted for the different components, the dose-response curve on which the attribution of scores is based, etc.

It also needs to be considered that the interpretative approach, being based on algorithms that are intrinsically complex and providing an overall assessment of the nutritional value of a food, that cannot be understood by the consumers, does not include the purpose to improve their knowledge. Ultimately, summary labeling aims to help the consumer in making single choices between different foods, but not to learn how to compose an overall healthy diet. Informative systems based on labeling Reference Intakes, on the opposite, provide all the numerical data (absolute amounts and percentage levels) needed to assess the energy and nutrient (fat, saturated fat, sugar, salt) content of one portion (and of 100 g for energy) of different foods, in the context of the energy and nutrient content of the whole diet. The extensive literature available focusing (although with several methodological approaches) on the effects of different FOPL schemes on level of consumer understanding and on choices at time of purchase should be reconsidered in view of the differences highlighted. Moreover, it is important to underscore that far fewer studies have assessed a direct relationship between the use of different FOPL under real life condition and the overall composition of people's diets (14).

Due to the different nature of the various FOPL developed so far, hence, there is some debate about which characteristics an efficient labeling system should have to reach the main objective, e.g., helping people in making informed, conscious and healthier food choices. In particular, the debate regards the possibility that the specific characteristics of different FOPL (e.g., nutrient-specific vs. summary labels, interpretative vs. non interpretative labels) may differently impact on this objective.

The analysis of some application examples of the different FOPL systems may help to show the related criticalities.

## FOPL and nutritional characteristics

### Similar FOPL for products with different nutrient composition

It cannot be overlooked the great variability in terms of nutritional characteristics that can be detected among products belonging to the same food category, especially if the aim is to compare different food products in order to choose the healthiest one (15, 16). It is therefore difficult to hypothesize that the classification into two categories (healthy or less healthy) or even into a few categories (as is the case with Nutri-Score) may reflect and properly clarify such complex situation.

As regards summary labels such as Nutri-Score or Keyhole (as any other algorithm-based system), it is worth noting that the same score/label may be the result of different nutritional characteristics and that it does not provide information about the individual nutrients contained in the product (mostly sugars, salt, saturated fats), which may instead be relevant for specific categories of consumers. For instance, the energy and nutrient content of different sweet cakes obtaining the same Nutri-Score (D) are reported in Table 2: not surprisingly, a large variability of composition in terms of some nutrient content, such as sugar (from 3.9 to 36.0 g/100 g), fat (from 14.0 to 26.0 g/100 g) and saturates (from 2.5 to 14.0 g/100 g) is observed. A certain variability is also observed with regard to fiber content, ranging from 1 g/100 g of item 6 to 3.4 g/100 g for item 5. Since these differences are not detectable by considering only this synthetic (algorithm-based) type of FOPL, the information provided appear to be inadequate for people who want (or need) to choose products in order to limit their intake of saturates (for example because of a slightly elevated blood cholesterol level), or of sugar (if their glucose tolerance is impaired), or salt (for a mild increase in blood pressure) or to improve their fiber consumption (17).

**Table 2:** Energy and nutrient content per 100 g of 12 sweet snacks currently on the Italian market, selected for having the same calculated Nutri-Score (D).

	**Sweet cake 1**	**Sweet cake 2**	**Sweet cake 3**	**Sweet cake 4**	**Sweet cake 5**	**Sweet cake 6**	**Sweet cake 7**	**Sweet cake 8**	**Sweet cake 9**	**Sweet cake 10**	**Sweet cake 11**	**Sweet cake 12**
Energy (kJ)	1,495	1,641	1,796	1,618	1,741	1,642	1,933	1,657	1,709	1,728	1,764	1,734
Energy (kcal)	357	392	429	386	416	392	462	396	408	413	422	414
Fats (g)	14.0	16.0	20.7	21.0	21.0	16.0	26.0	16.0	19.0	20.0	21.3	20.0
Saturates (g)	2.5	5.4	2.9	9.1	8.0	4.3	3.5	8.8	8.1	8.5	14.0	9.1
Sugar (g)	29.0	21.0	28.0	3.9	15.0	36.0	31.0	24.0	22.0	22.0	17.0	22.0
Fiber (g)	3.0	1.7	1.3	2.5	3.4	1.0	1.5	1.9	2.1	2.0	1.9	2.1
Protein (g)	6.4	7.4	5.9	7.4	8.6	6.3	6.2	6.8	6.2	6.2	6.7	6.5
Salt (g)	0.72	0.44	0.64	0.71	0.75	0.46	0.70	0.42	0.57	0.57	0.44	0.56

### Different messages from different summary FOPL schemes

It is of interest to note that different synthetic FOPL such as Nutri-Score and Keyhole do not necessarily end up in comparable evaluation of individual products, implying that the information on which algorithms have been built are significantly (and conceptually) different. As an example, Table 3 shows nutrient information for four items of breakfast cereals. Between the two products (items 1 and 2) obtaining the same Nutri-Score A (even though the great differences in salt content, which is more than double in one than in the other and in the presence of whole grains which represent 100% of item 1 and are completely absent in item 2), only one matches the criteria needed to bear the Keyhole and can be identified as healthier than the other one. Similarly, of the two B-rated products, only item 3 gets the Keyhole logo, despite its higher sugar and saturated fat content. This observation suggests that, besides being synthetic and not highlighting data related to specific nutrients, the criteria used for the two algorithms largely differ, so that for instance the Keyhole logo can be obtained by products not deserving the best Nutri-Score. These differences may also indicate that the overall nutritional assessment of foods and diets is different in different European countries, highlighting the difficulty of identifying a single system shared at the EU level, as required by current legislation; moreover, they thus question the opportunity to base public information on general principia that are not yet completely shared among experts.

**Table 3:** Energy and nutrient content per 100 g of breakfast cereals sold on the Italian market and selected for having different calculated Nutri-Score and for having or not Keyhole.

	**Breakfast cereals 1**	**Breakfast cereals 2**	**Breakfast cereals 3**	**Breakfast cereals 4**
Energy (kJ)	1,573	1,558	1,555	1,604
Energy (kcal)	376	372	372	383
Fats (g)	1.0	1.4	1.7	0.7
Saturates (g)	0.2	0.2	0.5	0.2
Sugar (g)	0.3	6.2	10.8	6.6
Fiber (g)	5.0	7	7.5	3.2
Protein (g)	8.0	13	9.4	7.3
Salt (g)	1.0	0.4	0.95	1.0
WG* content (%)	100	0	58	0
Nutri-Score	A	A	B	B
Keyhole**	Yes	No	Yes	No

*WG: whole grains **General criteria for the assignment of the Keyhole label: less fat, healthier fat, less sugar, less salt, more fiber and wholegrain, more fruits and vegetables, no sweeteners (food additives), no novel foods with sweetening properties, no phytosterols/phytostanols or their esters, not on foodstuffs for children up to 36 months.

The differences in energy and nutrient contents among the different products are more easily identifiable with the use of NutrInform Battery FOPL, providing more detailed information on calories per 100 g of products and the content of energy and nutrients per portion, both as absolute amounts and as percentages of the daily reference amounts, namely Reference Intakes (the formerly Guideline Daily Amounts), defined for calories and nutrients. NutrInform Battery highlights that similar portions of the four breakfast cereals are not significantly different in terms of energy supply; however, it shows that item 3, although bearing the Keyhole logo, has the highest sugar content, which is in turn higher than that of item 4, even if obtains the same score (B) with the French FOPL system. However, an informative approach, such as that of NutrInform Battery, may be less effective for not educated consumers. On the other hand, Nutri-Score can, as an example, prevent consumers from purchasing nuts since they would have a C, although these items are associated with health protection.

### FOPL and the nutritional role of portion

Beside the potential issue due to the large variability of nutrient contents in products that may get the same score, another criticism concerns the reference amount of product to be considered for the FOPL definition: 100 g, as for Nutri-Score and Keyhole and the portion or sales unit for products sold in single portion, as for NutrInform Battery as well as Traffic Lights. On the one hand, the adoption of systems based on 100 g would make it possible to compare products that are currently marketed in sales units of different sizes; on the other hand, however, it is worth noting that in most cases, the portion, or the quantity of food to be consumed for each eating occasion, is largely different from 100 g. As a consequence, the information provided for 100 g of product, which does not reflect the real absolute intake of energy and nutrients with a portion can indeed mislead the consumer.

As an example, the messages implied in FOPL based on 100 g of food products that should be consumed in much lower quantities for each eating occasion can result in the same products being perceived by consumers as more unfavorable or more favorable than their real nutritional role within the overall diet. This aspect cannot be disregarded, since it has been shown that the use of nutrition claims which are recognized as particularly favorable by consumers can increase their perceived healthfulness (18) and promote their purchase and consumption, especially in overweight subjects (19), and that FOP labeling had significantly stronger influence than nutrition claims on consumers' perceptions (20).

For instance, this is the case of cookies, whose standard portion size, to be consumed as part of a balanced diet, is set by Italian guidelines at 30 g. Despite the quite large variability in terms of energy and nutrient content observed when data are expressed per 100 g of products of different types of cookies currently available on the Italian market (Table 4), both the energy and nutrient supply appears to be less important when data are expressed for single portion. Moreover, the most relevant differences are not necessarily those considered to be important within the algorithm used for Nutri-Score calculation. As an example, the main differences observed between item 1 and item 2, obtaining an A and a B, respectively, with Nutri-Score, concern the content of sugar (lower in item 1) and that of fiber (higher in item 2); however, if considering one portion of products, the main aspect appears to be the contribution to the daily intake in terms of fiber of item 2, supplying about 20% of the total recommended intake, while sugar contained in the same item corresponds to only about one tenth of the maximum level set for total sugar. On the other hand, the energy and nutrient amounts provided by one portion may be similar even between products that obtain different Nutri-Score, as for items 2 and 3, whose contribution in terms of saturated fat, sugar and salt is comparable, although corresponding to a B and a C, respectively. NutrInform Battery allows to appreciate the differences in sugar and saturated fat contents and consequently in terms of contribution to the total saturated and sugar contents of the daily diet; however, it disregards information on fiber, which can be reported on the back of pack label of products, according to Regulation (EU) n. 1169/2011. It is well known that portions are not unambiguously coded for, by now, and that therefore there is considerable discretion and variability in defining their size in different countries (21); nevertheless, the role that portions play in determining the nutritional effects of diets cannot be overlooked. In fact, a portion-based labeling would bring significant advantages to the consumers, allowing them to understand the role of each individual food (and specifically its energy and nutrient supply) to the total daily intake and helping them to compose an overall healthier and balanced diet, which may more likely be the result of combining portions of different foods rather than indefinite amounts of the foods themselves (22).

**Table 4:** Energy and nutrient content of cookie items sold on the Italian market, per 100 g with different calculated Nutri-Score, and per portion (30 g) with percentage contribution of each portion to Reference Intakes for energy and nutrients, as in NutrInform Battery.

	**Cookies 1**		**Cookies 2**		**Cookies 3**		**Cookies 4**		**Cookies 5**		**Cookies 6**		**Cookies 7**	
Nutri-Score	A		B		C		C		D		D		E	
	100 g		100 g		100 g		100 g		100 g		100 g		100 g	
Energy (kJ)	1,664		1,615		1,972		1,895		1,990		2,022		2,046	
Energy (kcal)	398		386		471		453		476		483		489	
Fats (g/100 g)	11,2		9,4		19,0		18,0		19,0		20,8		21,7	
Saturates (g)	1.2		1.1		1.2		2.0		6.2		9.1		11.2	
Sugar (g)	1.8		19.2		20		20		23		26		25.5	
Fiber (g)	6.0		14.8		6.5		11		3.5		3.8		2.0	
Protein (g)	9.0		6.7		7.6		8.0		7.9		6.1		7.0	
Salt (g)	0.85		0.22		0.7		1.0		0.4		0.375		0.45	
	30 g	RI %*	30 g	RI %*	30 g	RI %*	30 g	RI %*	30 g	RI %*	30 g	RI %*	30 g	RI %*
Energy (kJ)	499		485		592		569		597		607		614	
Energy (kcal)	119	6	116	6	141	7	136	7	143	7	145	7	147	7
Fats (g)	3.4	5	2.8	4	5.7	7	5,4	8	5.7	8	6.2	9	6.5	9
Saturates (g)	0.4	2	0.3	2	0.4	2	0.6	3	1.9	10	2.7	14	3.4	17
Sugar (g)	0.5	1	5.8	4	6.0	7	6.0	7	6.9	8	7.8	9	7.7	8
Fiber (g)	1.8		4.4		2.0		3.3		1.1		1.1		0.6	
Protein (g)	2.7		2.0		2.3		2.4		2.4		1.8		2.1	
Salt (g)	0.3	5	0.1	2	0.2	3	0.3	5	0.1	2	0.1	2	0.1	2

^*^RI %: percentage of Reference Intakes (Energy: 2,000 kcal; Fats: 70 g; Saturates: 20 g; Sugar: 90 g; Salt: 6 g) set out Regulation (EU) n. 1169/2011.

The importance of considering the portion size even in FOPL can be further demonstrated by comparing a summary FOPL and the nutrient content of food products that are typically consumed as single pieces (e.g., pizza, flatbreads, sweet cakes) and for which different portions are currently available on the market. In fact, in all this cases, as the Nutri-Score, which is easier to understand compared to nutrient specific FOPL, is independent of the amount of product that is actually consumed (i.e., the portion), it can communicate misleading messages to the consumers, who are led to think that a green labeled product marketed in a larger portion may be nutritionally better than a red labeled product marketed in smaller portions in the same category. This can be for instance the case of flatbreads that are currently sold in single-portion packs ranging from 60 to 120 g, which can result in a very different energy and nutrient content per consumption unit. The comparison of 4 different flatbreads selected for having different Nutri-Score (from A to D) and that can be theoretically sold in different amounts such as 75 g, 100 g and 120 g (which are actually available in the Italian market) (Table 5) shows that the serving size may deeply affect the net intake of energy and nutrients with each flatbread, despite an overall increase in energy and nutrient contents per 100 g, as the Nutri-Score rises from A to D. As a result, for instance the net content of energy and “negative” nutrients (e.g., salt, saturated fat) in 120 g of flatbread with the most favorable Nutri-Score (A) may be higher than that assessed in 75 g of the product with the less favorable Nutri-Score (D). On the other hand, comparison of NutrInform Battery FOPL calculated for one portion of each product allows to assess the large differences in terms of energy and nutrient supply as absolute values and especially as percentage of the reference daily intakes: for instance, the lowest energy intake is associated to one portion of both items 1 and 4, which obtain Nutri-Score A and B, respectively; both items 1 and 7 provide the lowest amount of fat (9%), even if obtain A and C, respectively with Nutri-Score; as regards salt, four different products (items 3, 9, 11, 12) provide more than 30% of the daily reference intake per portion, even if obtaining different Nutri-Score (A, C, D and D).

**Table 5:** Energy and nutrient content per serving (75, 100, and 120 g), in different flatbread items with different Nutri-Score sold on the Italian market, reported as absolute values and as percentage of the Reference Intakes, as in NutrInform Battery (Legend: svg, serving).

	**Flatbread 1**			**Flatbread 2**			**Flatbread 3**			**Flatbread 4**		
Nutri-Score	A			B			C			D		
Serving (g)	75	100	120	75	100	120	75	100	120	75	100	120
Energy (kJ)	946	1,261	1,513	949	1,265	1,518	980	1,306	1,567	995	1,327	1,592
Energy (kcal)	226	301	362	227	302	363	234	312	375	238	317	380
Fats (g)	6.5	8.6	10.3	6.9	9.2	11.0	6.5	8.6	10.3	7.4	9.8	11.8
Saturates (g)	0.8	1.0	1.2	1.1	1.5	1.8	0.6	0.8	1.0	2.8	3.7	4.4
Sugar (g)	1.5	2.0	2.4	1.4	1.9	2.3	2.3	3.0	3.6	1.1	1.5	1.8
Fiber (g)	4.6	6.1	7.3	2.7	3.6	4.3	1.8	2.4	2.9	1.5	2.0	2.4
Protein (g)	6.5	8.7	10.4	6.5	8.7	10.4	6.5	8.7	10.4	5.9	7.9	9.5
Salt (g)	1.1	1.5	1.8	0.9	1.2	1.4	1.2	1.6	1.9	1.4	1.9	2.3
RI %*												
Energy	11	15	18	11	15	18	12	16	19	12	16	19
Fats	9	12	15	10	13	16	9	12	15	11	14	17
Saturates	4	5	6	6	8	9	3	4	5	14	19	22
Sugar	2	2	3	2	2	3	3	3	4	1	2	2
Salt	18	25	30	15	20	23	20	27	32	23	32	38

*RI %: percentage of Reference Intakes (Energy: 2,000 kcal; Fats: 70 g; Saturates: 20 g; Sugar: 90 g; Salt: 6 g) set out in Regulation (EU) n. 1169/2011.

These data demonstrate the importance of portion size in determining the absolute energy and nutrient content, and the contribution of the food product to the whole diet, as highlighted by the EU Regulation. Therefore, this aspect cannot be disregarded in the definition of a FOPL, in order not to mislead the consumer and to help him or her composing an overall healthier diet.

### How can FOPL application promote reformulation of food products?

Another aim of FOPL is to encourage the reformulation of food products by manufacturers. When interpretative systems are used, the goal of the reformulation is to move foods toward more favorable scores (23, 24).

Since different strategies are often simultaneously put in place and factors such as general market developments may affect the way the food companies change their products, it appears to be difficult to investigate the isolated effect of FOPL on reformulation. However, the association between FOPL use and the reformulation rate has been the subject of some recent research performed in different countries, with contrasting results. For instance, the analysis of the composition of 4,343 products with the Dutch Choices Logo over 10 years in the Netherlands demonstrated a general propensity to reformulate products to achieve a healthier nutrient composition in the same period, even though the degree of reformulation differed per product category and per nutrient (25). Indeed, total fat and sodium contents were significantly reduced in most products, whereas changes in energy, saturated fatty acids, added sugar and fiber were less consistent among categories (25).

Attempts to investigate the impact of FOP on reformulation have been also done in Australia and New Zealand. In Australia, where the Health Star Rating (HSR) - a summary FOPL system that rates the overall nutritional profile of packaged foods by assigning from $\frac{1}{2}$ a star to 5 stars - has been implemented, reformulation of packaged food products for children, that were available in 2013, occurred in 100% of HSR-labeled-products in comparison to 61.3% of non-HSR labeled products (26). However, the authors reported that only one-third of new products in the market were classified as “healthy,” so casting doubts on the idea that the HSR has actually stimulated the development of healthier food. Even in New Zealand, reformulation of HSR-labeled products before and after adoption of HSR (i.e., 2014 and 2016) was greater than that of non-HSR-labeled products over the same period, with greater energy and sodium reduction in HSR products than in non-HSR products (−1.5 vs. −0.4% for energy, and −4.6 vs. −3.1% for sodium) (22). However, caution should be taken in interpreting these results, due to the small number of products displaying HSR graphic labels (5.3% of packaged food and beverage products surveyed in 2016).

In Belgium, a significant reformulation of breakfast cereal products occurred between 2017 and 2018 in anticipation of the implementation of the Nutri-Score FOPL, with reductions in the content of total sugar (−5%; *p* < 0.001) and sodium (−20%; *p* = 0.002) and increases in fiber (+3%; *p* = 0.012) and proteins (+2%; *p* = 0.002) (27). However, the authors stated that it is difficult to attribute these changes (all below 5%, except for salt reduction) exclusively to the introduction of the Nutri-Score, as other commitments by manufacturers were ongoing during that time in Belgium that could have led to a product reformulation. A similar minimal reformulation (reductions in selected nutrient content below 5%) of food and beverage products was reported in Chile, 1 year before the implementation, in 2016, of the FOP warning labels for products high in sodium, total sugars, saturated fats and/or total energy (28). The Authors even reported some increases in critical nutrient and energy content of up to more than 5%.

The current evidence from studies evaluating the impact of food reformulation on nutrient intake as well as on food choices and health status was recently reviewed (29). About 3/4 of the 26 studies included in this analysis found positive results; however, most of them focused on the impact on the intake of salt (*n* = 20) and trans fatty acids (*n* = 5), and only one investigated the impact on whole grain consumption, while other nutrients of potential interest (e.g., sugar) and energy were not considered. Intriguingly, different results were observed based on the proxy of nutrient intake used in the different investigations. For instance, the positive impact on salt was greater when measured as salt purchased compared to salt intake measured using the 24 h urinary excretion. Another aspect pointed out by the authors is that, in reformulation, the reduction in a macronutrient content is usually obtained by an isocaloric substitution with another macronutrient, thus resulting in unchanged total energy density. Moreover, the very low quality of the available evidence in this field, mostly drawn on modeling studies, was underlined in a review of 16 studies investigating the impact of food product reformulation on sugar content (30).

For a throughout evaluation of the potential impact of FOPL on reformulation, it is worth highlighting that the feasibility of reformulation strictly depends on the type and characteristics of food products. On one hand, the reformulation is difficult for food products with specific formulations such as biscuits, cakes, or breads, in which it can also impact on technological, rheological or sensory properties. On the other hand, the nutrient composition, or the related summary FOPL, such as Nutri-Score, can be easily improved for many products through the addition of specific ingredients. This is for instance the case of pizza, in which the addition of vegetables can be effective in improving Nutri-Score from C to B (Table 6). However, the impact of reformulation of this kind of pizza on its nutrient composition and on the contribution to the daily intake in terms of calories and nutrients appears to be negligible, as shown by the NutrInform Battery label calculated for the two products with Nutri-Score B and A, respectively.

**Table 6:** Energy and nutrient content per 100 g and per portion^*^ of different formulated pizza sold on the Italian market, their Nutri-Score and percentage contribution of each portion declared by the manufacturer to Reference Intakes (RI %) (as in NutrInform Battery).

	**Pizza margherita**		**Pizza with vegetables**		**Pizza with vegetables** **(reformulated)**	
**Nutri-Score**	C		B		A	
	100 g		100 g		100 g	
**Energy (kJ)**	1001		767		767	
**Energy (kcal)**	239		183		183	
**Fats (g)**	9		6.6		6.6	
**Saturates (g)**	4.1		2.3		2.3	
**Sugar (g)**	3.6		3.7		3.7	
**Fiber (g)**	1.5		1.8		1.96	
**Protein (g)**	11		6.9		6.9	
**Salt (g)**	0.88		0.81		0.65	
**Na (calculated) (mg)**	352		324		260	
Fruit &Veg**(%)	23.9		42.9		42.9	
	150 g*	RI %***	190 g*	RI %***	190 g*	RI %***
**Energy (kcal)**	369	18	348	17	348	17
**Fats (g)**	14	19	13	18	13	18
**Saturates (g)**	6.2	31	4.4	22	4.4	22
**Sugar (g)**	5.4	6	7	8	7	8
**Salt (g)**	1.3	22	1.5	25	1.2	20

^*^ Portion declared by the manufacturer.^**^Fruits, vegetables, pulses, nuts, and rapeseed, walnut and olive oils.^***^RI %: percentage of Reference Intakes (Energy: 2000 kcal; Fats: 70 g; Saturates: 20 g; Sugar: 90 g; Salt: 6 g) set out in Regulation (UE) 1169/2011.

Attention should be also paid to the fact that, to improve interpretative FOPL such as Nutri-Score, reformulation could be minimal and made for the sole purpose of getting the product a higher quality score with minimal if any improvement of its nutritional value. For instance, as shown in Table 6, a vegetable pizza with Nutri-Score B can be further improved to A by reducing salt by 0.15 g per 100 g of products and with the addition of 0.15 g fiber, which has a very limited nutritional relevance.

The threshold system typical of interpretative, algorithm based FOPL could, in other words, indirectly facilitate (and promote) the reformulation of products with levels of single nutrients close to (just above or just below) the thresholds set; in these products small modifications of the composition, if they allow the decisional thresholds to be exceeded in the desired direction, can lead to a favorable reclassification. This opportunity is intuitively more complex to exploit for products whose composition is far from the thresholds and which should be drastically reformulated, with a high impact on sensory characteristics: with the potentially paradoxical consequence that products with greater nutritional criticality will not be reformulated, while those with small deviations from the proposed model will be.

Moreover, it is noteworthy that the framework for product reformulation should integrate nutrition and health but also food technologies, consumer science, legislation, economics and other disciplines (31). In this context, an emblematic case can be the call to action for replacing of palm oil, which was widely used in the past as cooking oil as well as food ingredient in baked products, due to its low cost, specific technological features, and industrial applications, with the aim to avoid negative effects on human and planet health potentially associated to the use of this vegetable fat rich in saturates (32). This approach would result in the replacement with unsaturated fats, allowing a more favorable fat composition of reformulated products. However, alternatives can have potential drawbacks mainly for technological reasons, for instance, faster oxidation and rancidity, and consequently shelf-life reduction. In many cases reformulation resulted to be technically unfeasible or required a large research and development effort by the food industries to develop low-cost alternatives (33, 34). In particular, in the context of a strategy aimed at encouraging the adoption of overall healthier diets, the economic implications of the reformulation of food products cannot be neglected, since they can differently affect food companies and possibly the availability of selected foodstuffs (35). As for the portion-based informative FOPL systems, the incentive for reformulation could essentially involve the reduction of portion size, which has been described as an efficient strategy to allow the consumption of adequate amounts of several foods, or at least the adaptation of the size of single-portion packs to the reference portions, and greater attention to size of multi-portion packs, to facilitate the consumer in using the most appropriate quantity of food (36). In this regard, it should be emphasized that the definition of reference portions, based on nutritional guidelines, for the different product categories is crucial, also to allow the correct comparison of the nutritional characteristics of foods belonging to the same category.

## Main conclusive considerations

The adoption of different FOPL systems, i.e., interpretative or informative, provides completely different information to consumers. The actual purpose of FOPL, defined by the European regulation (that is, to facilitate consumer understanding of the contribution or importance of the food to the energy and nutrient content of a diet) should be central in the choice of the scheme to be adopted.

Informative systems are more complex and require greater attention from the consumer; however, they are characterized by the transparency of the information conveyed and the educational function (37). NutrInform Battery, being based on reference portions, is proposed as a tool to help people understand the quantity of each food to be consumed as part of a balanced diet.

On the other hand, summary FOPL provide, in a simplified way, information which wants to be user-friendly; however, the use of algorithms to calculate the scores on which they are based are not free of critical aspects. As shown by the examples analyzed in this paper, each value can be the result of multiple combinations of parameters; moreover, the use of thresholds can produce different scores for products which have very similar nutrient composition and nutritional value. In both cases, the message is not clear: in the first one, two different products can be perceived as similar and alternatives, while in the second case, one product can be wrongly recognized as better than the other.

Moreover, advances in nutrition knowledge have led to the awareness of the importance of multiple factors underlying the interaction between diet and individual health, giving rise to the concept of personalized nutrition (or precision nutrition), which can justify the different effects observed for specific nutrients or diet components (e.g., saturated fat, or salt) in different population groups (38). From a public health point of view, it cannot be overlooked that products with different nutrition composition, which obtain the same summary FOPL, may have different effects on health of people according to individual characteristics, physiological/pathological conditions, and nutritional requirements. People who need to keep under control the intake of calories or that of a specific nutrient (i.e., large part of the general adult population) will not be helped by the application of a summary FOPL in the choice of foods suitable to build a healthy, adequate diet (39). Conversely, the presence of a positive or a negative message on the food package could be misleading for most of them, giving rise to food choices which are not necessarily healthier and can be potentially unfavorable for their health (18). Furthermore, the more recent scientific evidence confirms the complexity of the relationship between diet and health, suggesting that other aspects beyond the nutrient composition can influence the effects of the diet on human health: the presence of minor but biologically active components (for example polyphenols in vegetables, chocolate, tea and coffee) and fiber (in whole grains compared to refined grains), the glycemic index, some production processes (such as for example the fermentation of milk that gives rise to yogurt with partly different properties, or the transformation of meat), the structure of the matrix (as evidenced by the different role of saturated fats in milk derivatives). Such differences are very difficult to be accounted for in interpretative systems, since they are quite difficult to be included in the underlying algorithms (although some attempts have been made, i.e., for cheese in Nutri-Score); informative systems, on the other hand, could foresee a sort of add-on nutrition information, that could be object of a specific communication to the public.

Furthermore, an extensive literature in the field of behavioral economics shows that effectiveness, understanding, and acceptability of FOPL could be affected by other factors not strictly related to the nutritional aspects (e.g., economic and psychological factors), which could be also taken into account in defining the most suitable approach.

It is also worth underlining that the literature strongly supports the effectiveness of eating patterns based on a variety of foods (as well as on a healthy lifestyle), which are overall favorable for health, like the Mediterranean diet (40). The communication of the contribution of a single food to the overall diet, which is required by the European regulation, must include the amount of food that is actually consumed. In fact, nutrient intake is the result of the nutrient content of each food, the portion consumed and the frequency of consumption. Therefore, the presence of the simplified nutrition label on the front of pack of food products cannot disregard the concept of standard portion. In this context, it is important to underscore that the definition of standard portions for the different food categories must be a prerogative of the institutions, and not of food companies, and it should be shared by the different Countries and used as a reference for the FOPL.

Another criticism, concerning FOPL based on thresholds, is the impossibility to objectively define levels of energy, nutrients and ingredients which can be considered low, adequate, or high. Any threshold or range proposed will in any case be arbitrary, since even on the basis of all available evidence it will be impossible to define values shared by all the scientific community as absolute reference for all populations. Moreover, while it is quite obvious that nutrient values “just over” or “just below” have essentially the same nutritional value, the threshold system will convey the consumer, in such conditions, significantly or even completely different messages.

Some criticism may also concern the labeling Reference Intakes for energy and nutrients, on which the information delivered by the informative FOPL systems are based, which have been reviewed and defined by the experts of the EFSA Panel on Dietetic Products, Nutrition and Allergies, on a request from the European Commission, “to enable the nutrient content of a food product (per 100 g, per 100 ml, or per portion) to be expressed as a percentage of a typical recommended daily intake (adults)” and to allow “comparison of the nutritional values of food products” (41). Even if, according to the Authors, they have been derived “from science-based nutrient intake recommendations established by national and international authorities, which are based on evidence of relationships between intake and the risk of obesity and/or diet-related diseases (e.g., cardiovascular disease, diabetes mellitus, dental caries),” they must be considered only for labeling and distinguished from dietary reference values established for the different population groups. As a consequence, the selection of “value to take is not a scientific decision, but a management decision to be taken after careful consideration of all implications,” as stated by EFSA (42). However, as the reference values for the different population groups, even the levels indicated for energy and nutrients as labeling Reference Intakes should be periodically revised and possibly modified according to the indications of experts and health institutions.

About the possible reformulation of food products to obtain better scores, it should be considered that different kinds of foods have a specific nutrient composition and contain different amounts of nutrients by nature.

Interpretative FOPL, moreover, aim to synthesize a food composition in terms of different nutrients (e.g., saturated fats, salt, fiber), and are consequently forced to overlook the different role of the various nutrients in different foods (e.g., saturated fats in dairy and in meats). Even the adoption of different nutrient thresholds for different food categories, on the other hand, would involve some critical issues, mainly due to the role that both portions and frequency of consumption play in determining the impact of foods on the whole diet composition.

Furthermore, algorithms on which interpretative FOPL are based need to set criteria for the nutritional equivalence of different nutrients and ingredients, which are necessarily characterized by a large discretion: as an example, within Nutri-Score calculation, the same negative value (1 negative point) is attributed to one of these different conditions: energy higher than 335 kJ/100 g, saturated fat higher than 1 g/100 g or sugar higher than 4.5 g/100 g or salt higher than 90 mg/g. The equivalence of such thresholds is difficult to support in an evidence-based context.

Finally, the overall evaluation given by interpretative FOPL does not consider some nutritional aspects, that are relevant in terms of the relationship between nutrition and health, such as the high content of unsaturated fats in foods penalized by the high caloric intake (such as canned fatty fish), or the role of some products penalized for the sugar content as sources of polyphenolic compounds (such as dark chocolate). In the specific case of chocolate, the contribution of polyphenols is important for quantities of consumption compatible with a balanced diet, which are well below the 100 g, on which the sugar and fat content is instead evaluated.

The issue of selecting the data to be included in the calculation and the lack of transparency of the data provided to the consumer (i.e., the results of the algorithm) is overcome by informative FOPL, which merely provide precise numerical information without claiming to give an overall assessment of the food product.

As regards informative FOPL system, it should be considered that transparency and clarity of information, which are its strengths, could represent a limit. In fact, they require the consumer to be previously and adequately informed and instructed to understand and use the information provided by the FOPL in everyday life. Indeed, the centrality of education is highlighted by health institutions even to allow the general population to make proper food choices (43).

Furthermore, it is worth considering that there is a large literature in the field of behavioral economics that analyzes the effectiveness, understanding and acceptability of different FOPL, involving factors that are not strictly limited to nutritional aspects (e.g., economic and psychological), the evaluation of which, however, does not fall within the scope of this analysis.

In conclusion, it can be observed that the interpretative FOPL must necessarily be based on algorithms that summarize in a single score the information relating to different nutrients, or to other characteristics of a food (such as the energy content or the presence of selected ingredients). It follows that the ease of interpretation of the single score may be detrimental to the accuracy of the overall evaluation of a food, with a series of critical issues, as demonstrated by some examples which have been presented and discussed.

Whether such ease of interpretation of summary FOPL systems can compensate for the inaccuracies deriving from their use is not known and should be ascertained by means of adequate experimental studies.

Informative systems, on the opposite, provide the consumer with a less immediate message, and need to be supported by educational campaigns providing the information necessary to understand how to use the information obtained in order to combine foods and to build up a balanced dietary pattern; especially if based on portion sizes, they may actually help in pursuing this essential goal.

## Author contributions

All authors contributed to conceptualization, writing, reviewing and editing of the manuscript, and have read and approved the submitted version of the manuscript.

## Funding

Open access publication fees are supported by Nutrition Foundation of Italy.

## Conflict of interest

Authors AP and FM are, respectively, President and Scientific Director at NFI, a non-profit organization partially supported by 18 food companies. The remaining authors declare that the research was conducted in the absence of any commercial or financial relationships that could be construed as a potential conflict of interest.

## Publisher's note

All claims expressed in this article are solely those of the authors and do not necessarily represent those of their affiliated organizations, or those of the publisher, the editors and the reviewers. Any product that may be evaluated in this article, or claim that may be made by its manufacturer, is not guaranteed or endorsed by the publisher.
